# EPSPs Measured in Proximal Dendritic Spines of Cortical Pyramidal Neurons^1^,^2^,^3^

**DOI:** 10.1523/ENEURO.0050-15.2016

**Published:** 2016-05-12

**Authors:** Corey D. Acker, Erika Hoyos, Leslie M. Loew

**Affiliations:** 1 Richard D. Berlin Center for Cell Analysis and Modeling, University of Connecticut Health Center, Farmington, Connecticut 06030

**Keywords:** two-photon, basal dendrites, dendritic spine, glutamate uncaging, synaptic integration, VSD

## Abstract

EPSPs occur when the neurotransmitter glutamate binds to postsynaptic receptors located on small pleomorphic membrane protrusions called dendritic spines. To transmit the synaptic signal, these potentials must travel through the spine neck and the dendritic tree to reach the soma. Due to their small size, the electrical behavior of spines and their ability to compartmentalize electrical signals has been very difficult to assess experimentally. In this study, we developed a method to perform simultaneous two-photon voltage-sensitive dye recording with two-photon glutamate uncaging in order to measure the characteristics (amplitude and duration) of uncaging-evoked EPSPs in single spines on the basal dendrites of L5 pyramidal neurons in acute brain slices from CD1 control mice. We were able to record uncaging-evoked spine potentials that resembled miniature EPSPs at the soma from a wide range of spine morphologies. In proximal spines, these potentials averaged 13.0 mV (range, 6.5–30.8 mV; *N* = 20) for an average somatic EPSP of 0.59 mV, whereas the mean attenuation ratio (spine/soma) was found to be 25.3. Durations of spine EPSP waveforms were found to be 11.7 ms on average. Modeling studies demonstrate the important role that spine neck resistance (*R*_neck_) plays in spine EPSP amplitudes. Simulations used to estimate *R*_neck_ by fits to voltage-sensitive dye measurements produced a mean of 179 MΩ (range, 23–420 MΩ; *N* = 19). Independent measurements based on fluorescence recovery after photobleaching of a cytosolic dye from spines of the same population of neurons produced a mean *R*_neck_ estimate of 204 MΩ (range, 52–521 MΩ; *N* = 34).

## Significance Statement

While an excitatory synaptic input may typically generate a ∼0.5 mV depolarization at the soma, the magnitude of depolarization at its point of origin, the dendritic spine, is a subject of debate. We developed optical methods to excite and measure excitatory potentials at the target spine on the dendrites of cortical pyramidal neurons. These potentials are typically smaller than some previous reports, but are still > 20 times larger on average in basal dendrite spines than at the soma. We also show evidence that the spine neck resistance is an important biophysical parameter controlling these elementary neuronal input signals. The results provide a requisite basis for further studies on how synaptic inputs drive local voltage-dependent processes and cellular responses.

## Introduction

Dendritic spines constitute the postsynaptic sites of excitatory synaptic input in many mammalian brain regions and play central roles in learning and behavior. They are typically <1-μm-diameter membrane protrusions connected to the dendrite through a spine neck that is variable in diameter and length (Harris et al., 1992). Previous studies have shown the important role that spines can play in the compartmentalization of synaptic biochemical signals, such as small GTPases and calcium (Yuste et al., 2000; Murakoshi et al., 2011). However, understanding the electrical behavior of spines has been extremely challenging because their small size makes them inaccessible to traditional electrophysiological methods. Because the postsynaptic responses to synaptic input, as well as the likelihood that a synapse will undergo long-term plasticity, will largely depend on the interplay between biochemical and electrical signaling within spines (Yuste, 2013), the electrophysiological characterization of spines will deepen our understanding of the fundamental events underlying neuronal function. Accordingly, there have been significant efforts to measure the electrical response to synaptic activation in spines. These include estimates inferred from calcium changes measured with fluorescent calcium indicators in single spines (Bloodgood et al., 2009; Harnett et al., 2012) and measurements of voltage sensitive dye (VSD) signals in spines following electrical stimulation of synaptic inputs (Palmer and Stuart, 2009) or glutamate uncaging (Popovic et al., 2015).

Our laboratory previously developed single-voxel two-photon VSD imaging to measure backpropagating action potentials (bAPs) directly from single spines in acute brain slices (Acker et al., 2011; Acker and Loew, 2013). In the present study, we combine this method with two-photon MNI-glutamate uncaging (Ellis-Davies, 2014) in order to measure the voltage responses in individual spines to uncaging-evoked EPSPs. As opposed to electrical stimulation of nearby axons, the two-photon uncaging approach assures that we can stimulate single spines. We measure the amplitude and dynamics of uncaging-evoked EPSPs in single spines on the basal dendrites of L5 pyramidal neurons from acute brain slices, which we abbreviate spEPSP. At the same time, we measure this uncaging-evoked EPSP in the soma with a whole-cell patch and designate this soEPSP. When soEPSPs are in the range of physiological miniature EPSPs (ie, <1 mV), the spEPSP amplitudes never exceed 31 mV (mean = 13.0 mV). The attenuation ratios of EPSPs in spines relative to their amplitude values measured in the soma were 25.3 ± 12.2.

The spine neck resistance is a crucial variable that not only controls the attenuation of synaptic potentials, but also the local amplitudes of synaptic potentials in spines (Rall, 1974; Gulledge et al., 2012). In hippocampus, experiments combining calcium imaging, uncaging, and pharmacology produced estimates of spine neck resistances (*R*_neck_) centered ∼500 MΩ, and typically between ∼400 and 600 MΩ (Harnett et al., 2012). Another laboratory working in the same brain region using high-resolution stimulated emission depletion imaging, but without functional calcium or voltage-imaging, estimated *R*_neck_ to be much smaller, typically 56 MΩ (Tonnesen et al., 2014). In this work, we estimate *R*_neck_ in cortical pyramidal cells using two independent methods, which provided similar results. Using a biophysical model and inputting our experimental measurements of spine and somatic EPSP values, we arrived at *R*_neck_ values with mean 179 ± 25 SE MΩ. Additionally, in 34 spines we performed fluorescence recovery after photobleaching (FRAP) of a cytosolic dye, together with a novel image processing method to calculate the spine head volume, to estimate the same parameter, *R*_neck_. Again, similar values were found (mean 204 ± 21 SE MΩ, not statistically distinguishable, Kolmogorov–Smirnov two-sample, *p* = 0.48).

## Materials and Methods

### Two-photon microscopy

Voltage-sensitive dye imaging and glutamate uncaging were performed on a custom two-photon microscope based on a previously described setup (Acker et al., 2011). One Chameleon Ultra II (Coherent) was used for long-wavelength excitation at 1060 nm, whereas a Chameleon XR (Coherent) was used for uncaging at 750 nm. Laser power was modulated with two EOMs (Electro-optic modulator 350-80LA-BK with 302RM Driver, Conoptics). A 900 nm LP (long-pass) dichroic (Thor Labs) and a 710 nm LP excitation filter (Chroma Technology) were used in the long and short wavelength, respectively, excitation light paths. Light paths were combined using a 900 nm LP dichroic (Thor Labs) and passed through a 700 nm LP dichroic (Chroma Technology) for excitation/emission separation inside a modified Zeiss Axioskop 2 FS mot upright microscope (Carl Zeiss AG) equipped with a 40 × 1.0 NA water-immersion objective lens. In an added, non-descanned epifluorescence pathway, one “green” emission channel used a 540/25 nm bandpass combined with a 655 nm SP (short-pass; both from Semrock) filter, whereas a “red” emission channel used a 680 nm SP filter (Semrock). Epifluorescence emission channels were separated by a 585 nm LP dichroic (Chroma Technology). Red fluorescence was also collected in trans-fluorescence pathway as previously described (Acker et al., 2011). Two *x*–*y* galvanometer (galvo; 3 mm on 6515H, with 671HP servos, Cambridge Technology) systems were used to separately control the positioning of the uncaging and recording lasers in a custom scan head. Laser scanning was controlled by ScanImage v3.8 (Vijay Iyer; Pologruto et al., 2003) with customizations necessary for control of two sets of galvos and “single-voxel” recordings (Acker et al., 2011). The focal depths of the VSD excitation and uncaging lasers, which had very different wavelengths (1060 and 750 nm), were matched by adjusting a telescope in the 750 nm excitation pathway (confirmed using red fluorescent beads).

### Electrophysiology and dye loading

CD1 mice (postnatal day 17–30 of either sex) were anesthetized by inhalation of isoflurane, and decapitated according to an animal protocol approved by the Center for Comparative Medicine, University of Connecticut Health Center. Coronal brain slices (300 μm thick) were cut from the frontal lobes anterior to genu of corpus callosum using a vibrating tissue slicer perfused with ice-cold oxygenated (95% O_2_/5% CO_2_) artificial (ACSF). ACSF contained the following (in mM): 127 NaCl, 25 NaHCO_3_, 25 d-glucose, 3.5 KCl, 1.25 NaH_2_PO_4_, 1 MgCl_2_, 2 CaCl_2_, pH 7.4, osmolarity 306. Slices were incubated in a submerged holding chamber in ACSF at 35°C for 25 min and subsequently maintained at room temperature (∼22°C). Somatic whole-cell recordings were made at room temperature in a recording chamber perfused with oxygenated ACSF prepared the day of the experiment. Whole-cell recordings were made from layer 5 (L5) pyramidal neurons within the ventral medial prefrontal cortex, including the prelimbic and infralimbic areas. L5 pyramidal neurons were visually identified using infrared differential interference contrast optics. Cells that were ∼35 μm deep from the surface of the slice were selected for patching to minimize scattering of emitted photons, and to optimize penetration of the MNI (4-methoxy-7-nitroindolinyl)-glutamate (MNI-glu; Tocris Bioscience). Whole-cell recording pipettes (9–12 MΩ) were tip filled with intracellular solution containing the following (in mM): 135 K-gluconate, 7 NaCl, 10 HEPES, 2 MgCl_2_, 2 Na_2_-ATP, 0.3 Na_2_-GTP, pH 7.2 adjusted with KOH (1 M), osmolarity 275. Pipettes were back-filled with intracellular solution containing 3 mm of voltage-sensitive dye di-2-AN(F)EPPTEA (Yan et al., 2012). Passive transfer of the VSD into the neuron was monitored at the soma by exciting the dye at 1060 nm (0.7 mW). As soon as the soma fluorescence was bright (usually after ∼10 min), the loading pipette was pulled out. The dye-filled neuron was left undisturbed for about 1 h to allow diffusion of the VSD throughout the dendritic arbor. After this, the neuron was repatched with a pipette containing dye-free intracellular solution. All recordings were made using a patch-clamp amplifier (Axopatch 200B, Axon Instruments) in current-clamp mode with voltage low-pass filtered at 2 kHz.

### Glutamate uncaging

MNI-glu (Tocris Bioscience) was applied through an extracellular pipette with a broken tip located 40 μm from the surface of the slice, close to the target dendrite. The pipette contained MNI-glu (15 mm) and AlexaFluor 488 (10 μm) dissolved in fresh ACSF. L5 pyramidal neurons filled with the VSD where visualized with two-photon excitation at 1060 nm, and bright proximal spines on basal dendrites that were well isolated from neighboring spines and the dendrite were targeted for uncaging. EPSPs at the soma were evoked by 0.5 ms uncaging pulses at 0.1 Hz. An interleaved protocol consisting of uncaging and control (no-uncaging) trials was used for noise analysis and determination of detection thresholds. MNI-glu photolysis was done at 750 nm to minimize the bleedthrough of the uncaging laser into the VSD channel, using average powers of 30–35 mW measured after the objective. The experiment was terminated if any signs of photo-damage were observed, such as changes in the spine morphology often associated with persistent depolarization at the soma.

### Data analysis

All the data analysis was done using custom code written in MATLAB. Single-voxel optical recordings of the VSD fluorescence (5 MHz sampling rate) in the spine were low-pass filtered with a frequency cutoff of 0.35 kHz (3rd order Chebyshev Type I) for uncaging and interleaved control trials. Raw data were first time-reversed before being passed to MATLAB’s *filter* routine in order to prevent corruption of EPSP onsets with filter start-up transients. This procedure was done after the 0.5 ms uncaging artifact was removed from the optical recordings. Data was finally passed through a 2.5 ms boxcar moving average in the forward direction. Typically, 7–30 uncaging and corresponding interleaved control trials were averaged. Individual uncaging optical records were only analyzed if a soEPSP could also be detected.

Optical recordings of backpropagating action potentials were low-passed filtered at 1 kHz for action potentials (5th order Chebyshev Type I). The MATLAB built in function *filtfilt* was used, which yields zero phase distortion of the original signal.

Photobleaching of VSD, along with a slow onset transient attributed to the EOM, was subtracted from the averaged sweep by fitting it to a product of two exponentials using the custom MATLAB scripts based on the *fminsearch* routine. After this, the standard deviation (SD) of data within a moving 5 ms window was used to estimate the noise level (σ_noise_ when converted to percentage Δ*F*/*F*), and from this, a detection threshold equal to 2.5 × σ_noise_ was determined (Palmer and Stuart, 2009). Optical spEPSPs that crossed this threshold following the uncaging pulse were fitted by an alpha-like function of the following form:

(1) $$\Delta \frac{F\left(t\right)}{F}=\;K\left(e^{-k_{2}t}-\;e^{-k_{1}t}\right).$$

Here, *K*, *k*_1_, and *k*_2_ are parameters that are optimized to fit the data, from which the amplitude and duration of the optical spEPSPs were estimated. Fits were obtained using similar MATLAB scripts as described above for baseline, photobleach subtraction. Amplitudes in percentage Δ*F*/*F* were then converted to millivolts using the calibration factor determined from backpropagating action potentials as described below.

In optical recordings of bAP in spines, control trials and trials with current injection were again alternated. Four to 10 sweeps or trials for both conditions were averaged. Trial-to-trial temporal jitter of bAP waveforms was eliminated using spike-triggered averaging (Acker and Antic, 2009) and signals were converted to percentage Δ*F*/*F* as described above.

Descriptive statistics were calculated in SAS 9.4 and MATLAB. Bootstrapping (400,000 samples) was used in computing confidence intervals for both *R*_neck_ datasets (Table 1). A Kolmogorov–Smirnov two-sample test (K–S) was used to test whether the two independent *R*_neck_ datasets (Table 1) could follow the same distribution. Because the *p* value of the K–S test was 0.48 (>0.05, not significant), the null hypothesis of following the same distribution could not be rejected. In other words, the possibility that the two sets follow the same distribution could not be excluded.

**Table 1.** Statistical table

**Analysis variable, *R*_neck_**								
**Set**	***N* Obs**	**Mean**	**SD**	**Median**	**Minimum**	**Maximum**	**Lower 95% CL for mean**	**Upper 95% CL for mean**
VSD measurements + model	19	179.4	109.1	210.2	22.9	419.6	132.1	227.7
FRAP experiments	34	204.3	125.3	161.1	51.8	520.6	164.8	247.6

### Estimating the spine neck resistance

*R*_neck_ was estimated based on the previously described (Svoboda et al., 1996) relationship between cytoplasmic resistivity and diffusion time constants in the spine:

(2) $$R_{\text{neck}}=\;R_{\text{a}}\;\times \;\tau_{\text{eq}}\;\times \;D_{\text{Alexa}488}/{\text{Vol}}_{\text{head}}.$$

Where *R*_a_ is the cytoplasmic resistivity, taken to be 150 Ω cm (Hu et al., 2009; Branco et al., 2010; Harnett et al., 2012); τ_eq_ is the equilibration time constant of AlexaFluor 488 in the spine head measured through the FRAP experiment; *D*_Alexa488_ is the diffusion coefficient of AlexaFluor 488 in the cytoplasm, 380 μm^2^/s (Nitsche et al., 2004); Vol_head_ is the volume of the spine head, which we measured from the *z*-stacks of the AlexaFluor 488 fluorescence excited at 770 nm.

### Image analysis and spine head volume

All distances and spine volumes were determined from the *z*-stacks of the AlexaFluor 488 fluorescence using ImageJ software. A 3D convolution method was used to calculate volumes of spine heads. The idea is to use a refined segmentation procedure to derive an initial guess at a 3D shape for the spine head. This shape is then systematically dilated and eroded to develop a series of structures of varying volumes. These structures are then each convolved with a 3D point spread function and the resulting set of blurred images are correlated to the actual two-photon 3D image of the spine to find the best match. The volume of the segmented image that produced the best blurred image is then used in Equation 2.

To implement this image processing and analysis procedure, the following steps were performed. The point spread function (PSF) for two-photon excitation at 770 nm was measured using the *z*-stacks of 170 nm subresolution fluorescent beads. The MetroloJ plugin of the Fiji application in ImageJ was used to find the full-width at half-maximum of the fluorescence profiles in the *x*, *y*, and *z*. The PSF half-widths were 0.422 μm in *x*, 0.526 μm in *y*, and 1.16 μm in *z*. The pixel size was 34.32 pixels/μm and 0.3 μm/frame. Using the Gaussian PSF 3D ImageJ plugin (http://www.optinav.com/Convolve_3D.htm), a Gaussian PSF was generated that had the same *x*-*y*-*z* dimensions as the experimental PSF. To find the spine shape, the *z*-stack of the spine head was segmented using the 3D segmentation tool available in Virtual Cell (www.vcell.org/vcell_software/login.html); this segmented stack was scaled to the ratios of the PSF half-widths: *x*-scale = 1, *y*-scale = 0.8, and *z*-scale: 0.36. Eroding or dilating the segmented-scaled *z*-stack yielded different spine sizes (but maintaining the same shape), each of which was convolved with the 3D Gaussian PSF using the Convolve 3D plugin in ImageJ. The optimal size resulted in a peak correlation coefficient equal to the dilution ratio between the spine and the parent dendrite. This dilution ratio (maximum fluorescence in the brightest region of the spine/maximum fluorescent in the brightest region in the parent dendrite) was measured from the maximum fluorescence projection, after the outliers were removed and the image was smoothed (a filter that replaces each pixel with the average of its 3 × 3 neighborhood) using ImageJ. The volume of the optimal source binary 3D image was measured using an analysis feature in Virtual Cell.

### Simulations

Simulations were performed using the NEURON 7.3 simulation environment (Hines and Carnevale, 1997), based on a morphologically realistic model of a L5 pyramidal neuron described previously (Mainen and Sejnowski, 1996). Variable time step settings used were as follows: atol, rtol, maxstep = 10^−5^, 10^−4^, 0.5 ms, respectively. The passive electrical properties of the model consisted of membrane capacitance, axial resistivity, and membrane resistivity of 0.75 μF cm^−2^, 150 Ω cm, and 30,000 Ω cm^2^, respectively. Dendrite morphology, including process lengths and diameters, was adjusted for spine membrane not explicitly included in the model, under the assumption that spines occur at a given average density are populated with active and passive channels as in the original publication. The resulting input resistance at the soma was 43 MΩ. The resting membrane potential was −70.35 mV. The active properties of the model were as previously described (Mainen and Sejnowski, 1996), and consisted of voltage-gated sodium, potassium, and calcium channels, and calcium-gated potassium channels. A single spine (diameter = 1 µm), with the same passive membrane properties as the dendrite, was attached to a basal dendrite through a spine neck (l = 1 µm). Spine neck length is arbitrary since neck diameter was varied to achieve desired neck resistances. In all simulations, the spine head and the spine neck were each treated as single compartments. A point process alpha-like conductance was added to the spine head to mimic the synaptic conductance (*G*_syn,_ reversal potential = −5 mV), which followed a dual exponential (rise and fall) time course with 0.5 and 4 ms time constants, respectively. The site of attachment was moved between 117 sites covering all basal branches except those <30 μm from the cell soma. At each site, a series of maximal synaptic conductances (*G*_syn_) and *R*_neck_ values were simulated while the somatic and local EPSPs were recorded along with the distance to the soma.

Simulation results were saved to file and analyzed off-line in MATLAB. After applying each *R*_neck_ value to spines at 117 locations throughout the basal dendrites, an average attenuation ratio (spine–soma) as a function of distance was determined via polynomial fits (see Fig. 7). Finally, for each experimental attenuation data point (distance, spine–soma attenuation), linear interpolation was used to determine the model’s *R*_neck_ value that coincided with that point. In this way, the biophysical model was used to estimate *R*_neck_ by determining the *R*_neck_ values consistent with the experimentally observed spine soma attenuation vs. distance data.

## Results

### Quantifying spine EPSPs with single voxel voltage-sensitive dye recording

**Figure 1.**
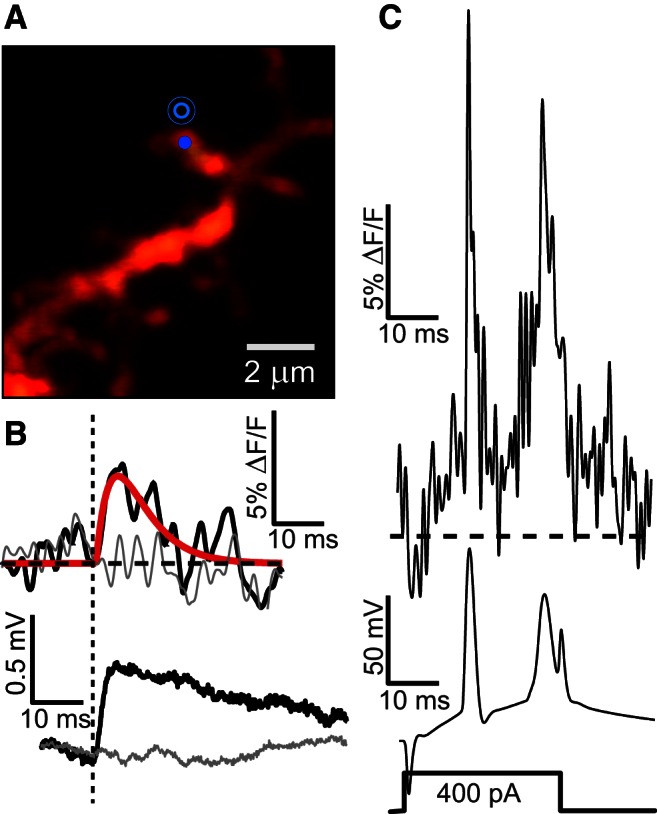
Detection of uncaging spEPSPs using a VSD. ***A***, *Z*-projection image of basal dendrite region, with spine of interest 92 μm from soma. On-spine recording, and peri-spine uncaging targets shown. ***B***, Simultaneous spine optical (top; spEPSP dark trace) and somatic whole-cell electrical (bottom; soEPSP dark trace, 0.53 mV) recordings of uncaging induced EPSPs. Averaged uncaging sweeps are superimposed on control sweeps (light traces). Red trace is dual-exponential fit to spEPSP. Average of *N* = 6 sweeps uncaging and control, σ_noise_ = 0.63%. Vertical dashed line indicates time of uncaging pulse (0.5 ms pulses). ***C***, Simultaneous optical (top; same spine) and somatic whole-cell electrical (bottom) recordings of APs (current step depicted below) used for calibration. Averages of *N* = 10 sweeps APs, 23.6% peak. Same scale bars apply to all optical recordings for ease of comparison.

To study the amplitude and dynamics of uncaging-evoked spEPSPs, we built a two-photon imaging system that allowed us to simultaneously perform two-photon MNI-glu uncaging and record fluorescent VSD responses from single spines, while electrically measuring soEPSPs through a whole-cell patch (Fig. 1). The VSD that we used in this study, di-2-AN(F)EPPTEA (aka PY3243) (Yan et al., 2012), is a fluorinated hemicyanine intracellular dye optimized for two-photon excitation. Previous work in our laboratory has shown that exciting this dye at 1060 nm with single-voxel excitation (stationary laser spot) typically results in single sweep signal-to-noise (S-N) ratios of 6 for optical recordings of bAP in spines from acute brain slices (Acker et al., 2011).

To optically measure spEPSPs, we first measured the bAP in the target spine and at the soma for calibration purposes (Fig. 1*C*), and then interleaved uncaging with non-uncaging or control trials (Fig. 1*B*). Control trials were used for noise estimates and baseline, photobleaching subtraction. Glutamate uncaging pulses were delivered at 0.1–0.3 Hz to avoid potentiation or depression of the postsynaptic response. Initially, the uncaging power was increased until a reliable soEPSP in a typical physiological range between 0.2 and 1 mV was observed from the electrical recording at the soma. To search for the optimum uncaging position leading to large, fast rising electrical responses for a given laser power, and presumably corresponding to the site of the postsynaptic density, the uncaging spot was positioned at various locations around the spine.

**Figure 2.**
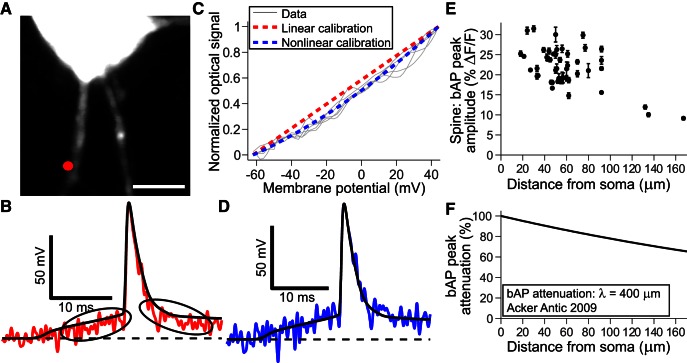
Calibration of the voltage dependence of spine Δ*F*/*F* using optical recordings in spines of bAPs. ***A***, Image of neuron loaded with VSD and a region of basal dendrites. Red dot shows juxtasomatic basal dendrite recording location. Scale bar, 5 μm. ***B***, Superimposed electrical action potential and optical bAP recordings (average of *N* = 8 sweeps) from juxtasomatic site showing reduced optical sensitivity at subthreshold potentials (circled). Peak optical sensitivity = 13.8% Δ*F*/*F*; 200 pA, 50 ms current injection. ***C***, Binning and plotting optical (peak normalized) versus electrical data shows nonlinear voltage-dependent VSD sensitivity (*N* = 5 trials, gray lines). Blue dashed line shows quadratic fit to sensitivity data, whereas red dashed line (shown for comparison) represents a linear relationship between rest and the peak of the AP. ***D***, Adjusting the optical data with nonlinear sensitivity calibration from ***C*** demonstrates accuracy in both subthreshold and superthreshold potential ranges. ***E***, Amplitude of the optical bAP in the spine (percentage Δ*F*/*F*) versus distance from soma for *N* = 48 spines across 32 different cells. ***F***, Attenuation of bAPs as a function of distance from soma in basal dendrites of L5 pyramidal neurons as previously reported using a VSD (length constant λ = 400 μm; Acker and Antic, 2009).

Calibration of optical signals in percentage Δ*F*/*F*, as shown in Figure 1*B*, to membrane potential in millivolts required three steps: (1) to correct a small but significant nonlinearity in the sensitivity of the VSD, (2) to determine the sensitivity of the VSD recordings in the particular spine of interest, and (3) to compensate for distance-dependent effects. Superimposing optical and electrical recordings of action potentials (APs) revealed that the dye response was less sensitive in the subthreshold voltage range compared with the sensitivity observed at the AP peak (Fig. 2*B*), which if not corrected for would lead to an underestimate of spEPSP amplitudes. Plotting the optical signal versus electrical signal at juxtasomatic sites (on basal dendrite <20 μm from the soma; Fig. 1*A*) showed a consistent, smooth curve that we fit with a simple, two-parameter quadratic function (Fig. 2*C*). Small signal sensitivity was 65% of the sensitivity at the AP peak. After correcting for the nonlinearity we found we could accurately reproduce AP waveforms in subthreshold to superthreshold regimes (Fig. 2*D*).

AP recordings were used to determine VSD sensitivity at individual spines. Use of bAPs to calibrate VSDs in spines was described previously (Palmer and Stuart, 2009). Figure 2*E* shows the Δ*F*/*F* amplitude from bAPs measured on 48 spines as a function of the distance of the spines from the soma. The scatter in the data show that individual spines exhibit different fluorescence sensitivities to potential. This is presumably due to different levels of background fluorescence from internal membranes (but not cytosolic dye, which is not fluorescent) and is the reason that calibration on a spine-to-spine basis with bAPs was necessary. More or less background fluorescence due to internal membrane staining leads to lower or higher sensitivities, respectively, by causing changes in *F* but not Δ*F*. The mean amplitude of the optical AP in the spine was 21.9 ± 4.9% Δ*F*/*F*, whereas the mean distance of the spines to the soma was 59.1 ± 28.9 μm (*N* = 48 spines from 32 cells).

Knowing both the optical signal amplitude and electrical signal amplitude of a bAP in a spine determines the dye’s sensitivity in that particular spine, which is the calibration factor for converting optical signals in Δ*F*/*F* to millivolts. However, the actual amplitude in millivolts is only known at the soma, and a correction must be applied according to the amount of attenuation that occurs during propagation to the spine. The average attenuation of the bAP’s amplitude with distance in the basal dendrites of L5 pyramidal neurons has been previously measured (Fig. 2*F*); using VSD imaging from basal dendrites within a similar developmental time window as the one in this study (postnatal day 17–30), Acker and Antic (2009) reported attenuation approximating a decaying exponential with a length constant (λ) of 400 μm. To minimize the effect of dendritic filtering and amplitude attenuation, we focused on proximal spines on basal dendrites; however, we did correct for the small attenuation of the bAP using the curve in Figure 2*F*.

The main challenge of measuring EPSPs in single spines is to achieve a high signal-to-noise ratio in the optical recordings. We performed a noise analysis that allowed us to establish a reliable detection threshold for an optical spEPSP. For this analysis, we relied on interleaved control trials, where no uncaging pulse was applied (see Materials and Methods; the photobleaching rate and noise between uncaging and control trials did not differ significantly). We established a detection threshold from the control trials that was equal to the mean VSD fluorescence plus 2.5 × the SD over a 5 ms running average (σ_noise_). Thus, in the uncaging trials, only transients that crossed this threshold and that occurred at the uncaging event were considered to be measurable optical spEPSPs. These spEPSPs were fit to an alpha-like function from which the amplitude and duration (half-width) were determined (Fig. 1B; Eq. 1).

**Figure 3.**
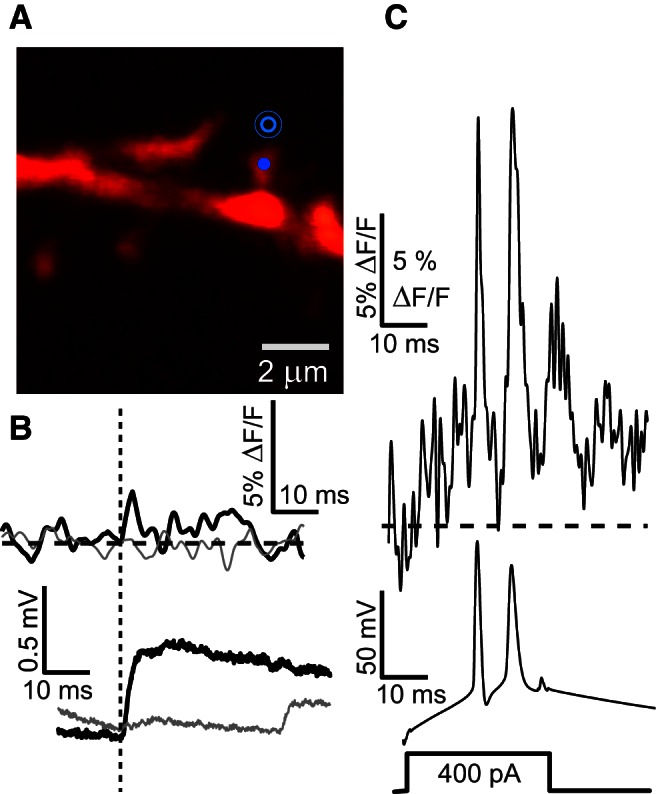
Uncaging responses in spines that are below the optical measurement threshold: clear optical bAP measurements serve as positive controls and for signal calibration. ***A***–***C***, Same as Figure 1 on a different dendrite with target spine 52 μm from soma. A clear soEPSP is seen in ***B*** (0.5 mV peak), but no clear optical spEPSP is seen in ***B*** (top; no fit is made), whereas at the same time bAPs are clearly measureable optically in ***C*** (top; serves as positive control). Averages of *N* = 10 sweeps APs, 18.3% peak. Average of *N* = 10 sweeps uncaging and control, σ_noise_ = 0.3%.

Our ability to measure bAPs in every spine allowed us to calibrate the VSD response as discussed above. Importantly, these optical bAP measurements also served as a positive control for each measurement, assuring that the VSD was working and that we could establish a minimum threshold for EPSP detection. Figure 3 displays an example of an experiment where the VSD sensitivity was normal based on the bAP signal, and where uncaging produced a soEPSP, but where the S–N for the spEPSP was below our 2.5 × σ_noise_ threshold for further analysis. For such optical spEPSP signals that were below the 2.5 × σ_noise_ detection threshold, this threshold was used as the minimum percentage Δ*F*/*F* reliably detectable. By converting 2.5 × σ_noise_ to voltage, we obtain the signal “resolution” of the experiment in millivolts. For example, we are able to assert that the spEPSP in Figure 3*B* with a σ_noise_ of 0.3% had to be <5.5 mV.

**Figure 4.**
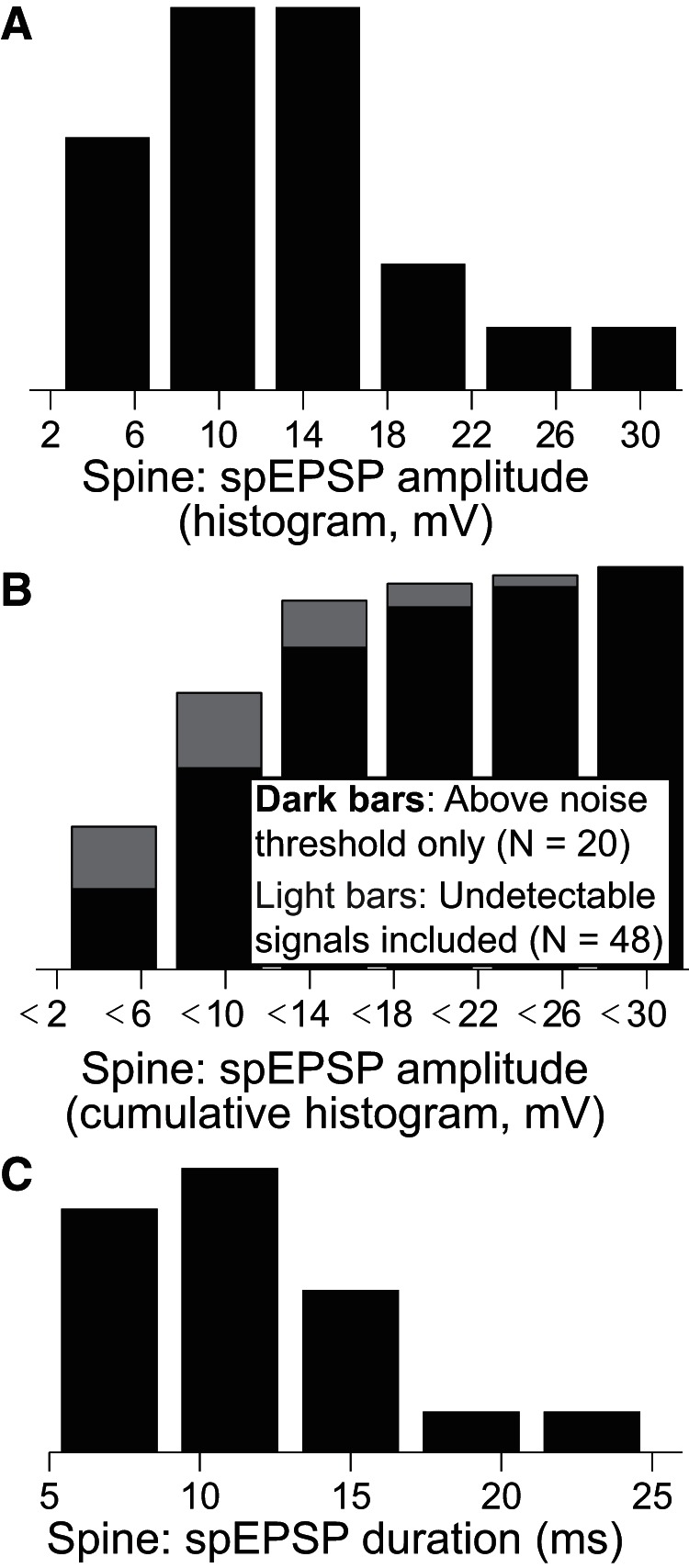
Amplitude and duration of uncaging-evoked spEPSPs. ***A***, Histogram of calibrated spEPSP amplitudes, produced from a dual-exponential fit, and taken only if fit amplitude exceeded noise levels (σ_noise_) by a factor of at least 2.5 (mean = 13.0 ± 6.7 mV, *N* = 20, calibrated using optical bAP measurements as shown in Fig. 2). ***B***, Cumulative histograms of spEPSP amplitudes. Dark bars correspond to supra-noise threshold data from ***A***. Additionally, noise thresholds from experiments with no measurable spEPSP (*N* = 28, but each still shows clear optical bAP from the spine and somatic EPSP as in Fig. 3) can be included because actual amplitudes must be less than this number (mean = 10.1 ± 5.6 mV, *N* = 48 total; light behind dark bars). ***C***, Histogram of the uncaging-evoked spEPSP half-widths (taken from fit curves, *N* = 19 spines, 1 point at 45 ms omitted; mean = 11.7 ms).

After calibration, the amplitude of the clearly measurable spEPSPs ranged from 6.5 to 30.8 mV with a mean of 13.0 mV (Fig. 4*A*; *N* = 20). These measureable, above threshold spEPSP amplitude values are plotted again in a cumulative histogram together with the upper limits of the spEPSPs that were below our 2.5 × σ_noise_ detection criterion in Figure 4*B*. These upper limits for spEPSP amplitudes fell within a range of 3.5–16.4 mV (*N* = 28); thus there are spEPSPs with amplitudes as low as ≤3.5 mV that still produce electrically measured soEPSPs. The duration of uncaging-evoked spEPSPs had a mean duration (full-width at half-maximum) of 11.7 ± 4.3 ms (Fig. 4*C*).

**Figure 5.**
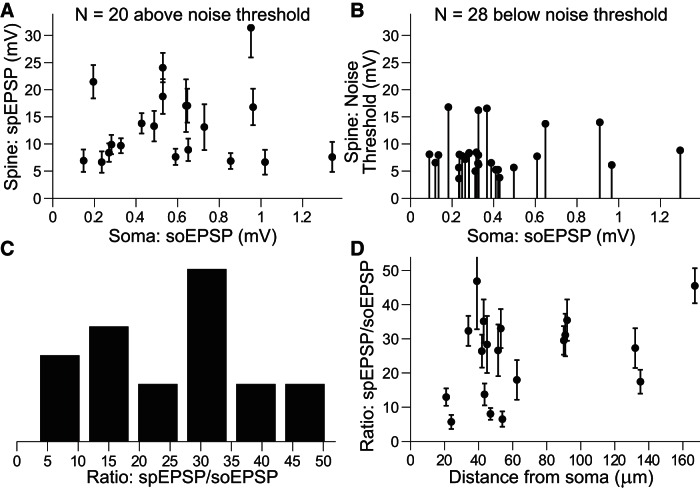
Uncaging-evoked EPSP amplitudes in the spine versus the soma. ***A***, Uncaging-evoked spEPSP amplitude versus the somatic soEPSP amplitude for spines with VSD signal above the detection threshold based on noise estimates (2.5 × σ_noise_, *N* = 20 spines). Error bars correspond to SD from time-series data, σ_noise_. ***B***, Stem plot showing the 2.5 × σ_noise_ threshold, which corresponds to the maximum voltage in the spine evoked by glutamate-uncaging, versus the somatic soEPSP for spines where the VSD signal was below the detection threshold (*N* = 28 spines). ***C***, Histogram of the ratio of spine to somatic EPSP amplitudes. The mean amplitude ratio was 25.3 ± 12.2. ***D***, Ratio of spine to somatic EPSP amplitudes versus distance from the soma.

Figure 5*A* shows the amplitude of the uncaging-evoked spEPSP versus the soEPSP (electrically measured somatic) for *N* = 20 spines calibrated using a bAP attenuation constant of 400 μm. There is no apparent correlation in Figure 5*A*. Likewise, Figure 5*B* shows no apparent relationship between soEPSP and the upper limits of spEPSP, when the spine data were below our detection threshold, although not surprisingly more points are clustered at lower soEPSP levels. The spine to soma amplitude ratio was on average 25.3 ± 12.2 (Fig. 5*C*) and did not have any apparent dependence on distance (Fig. 5*D*). This suggests that the voltage drop across the highly variable spine neck resistance may be the most important determinant of the EPSP attenuation between the spine and the soma at least for this range of spine distances.

### Estimating spine neck resistance by FRAP measurements from spines

**Figure 6.**
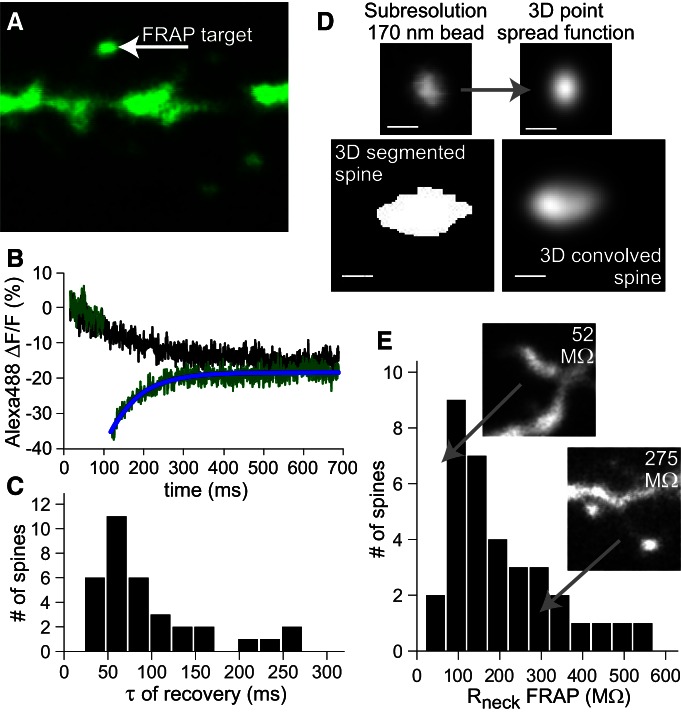
FRAP of an intracellular cytosolic dye was used to estimate *R*_neck_. ***A***, Sample image with spine targeted for photobleaching. Cells from the same population of neurons were filled with the cytosolic dye AlexaFluor 488. ***B***, After a 0.5 ms pulse of 770 nm excitation light, photobleaching of green fluorescence was apparent. Recovery approached control (black trace) and was fit to a single exponential (blue curve). ***C***, Histogram of fit exponential time constant (τ) values from *N* = 34 spines (mean = 95 ± 11 SE, median 72 ms). ***D***, Conversion to *R*_neck_ depends on the spine head volume (Eq. 2). We estimated spine head volume by 3D segmentation of *Z*-stack spine images (770 nm excitation) into binary images, eroding or dilating these binary 3D images to produce a series of sizes and then convolving with the 3D point spread function of the microscope obtained using 170 nm fluorescent beads. The resultant set of blurred images were then correlated with the experimental image to obtain the best fit. The source binary image which produced the best fit was then used to determine the spine head volume. ***E***, Histogram of *R*_neck_ values calculated from FRAP experiments (mean 204 ± 21 SE MΩ), with inset examples of large and small resistance examples.

To estimate the *R*_neck_ from the same dendritic region from neurons of the same population, we used two-photon FRAP of cytosolic AlexaFluor 488 (Fig. 6). FRAP experiments from sealed-end compartments allows the measurement of the AlexaFluor 488 recovery equilibration time constant (τ_eq_), which is determined by the diffusion barrier of the neck (Svoboda et al., 1996). Upon photobleaching, the AlexaFluor 488 dye has to diffuse from the dendrite through the neck to replenish the fluorescence in the spine; a neck that is narrow, long, or occluded with internal membranes will slow this process and would be mirrored by an increased *R*_neck_. We applied a 0.5 ms laser pulse at 770 nm on the target spine to bleach the AlexaFluor 488 (Fig. 6*B*). The recovery of the AlexaFluor 488 fluorescence was monitored in the green channel for 600–1,000 ms and was fit to a single exponential from which the τ_eq_ was calculated (Fig. 6*B*). The histogram of τ_eq_ for *n* = 34 spines (from multiple cells) is shown in Figure 6*C* (mean = 95 ± 11 SE, median 72 ms).

*R*_neck_ can be estimated from the relationship between τ_eq_, the AlexaFluor 488 diffusion coefficient, cytoplasmic resistivity and the spine head volume (Svoboda et al., 1996; see Materials and Methods; Eq. 2). Because the spine head is close to the resolution limit of the microscope, we developed an image-processing algorithm, which does not assume a given spine shape, to measure the spine volume from the AlexaFluor 488 *z*-stacks taken at 770 nm (see Materials and Methods). Briefly, it involves convolving the PSF of the microscope (Fig. 6*D*) with a set of 3D binary images derived from the original spine image to find the best fit to the original spine intensity distribution (Fink et al., 1998); the volume could then be determined from this optimal 3D binary image.

After converting the measurements of time constant and spine head volume values (Eq. 2) we arrived at a distribution of estimated *R*_neck_ values (*R*_neck_ FRAP; Fig. 6*E*) for 34 spines. The resulting distribution was skewed to smaller *R*_neck_ values, with a long tail to a maximum *R*_neck_ of 520 MΩ (min *R*_neck_ = 52 MΩ). Mean *R*_neck_ was found to be mean 204 ± 21 SE MΩ, with a median value of 161 MΩ.

### Estimating *R*_neck_ using biophysical models

We next considered whether the measured spEPSPs and soEPSPs were consistent with the *R*_neck_ values determined by our FRAP experiments. To explore this we turned to computational modeling.

**Figure 7.**
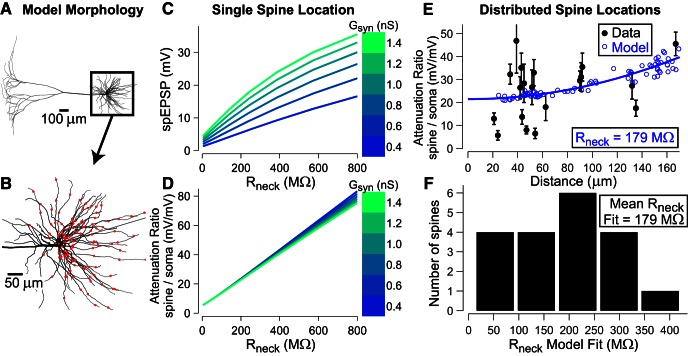
Estimating *R*_neck_ using biophysical models. ***A***, A morphologically realistic multicompartmental model of a L5 pyramidal neuron was used (Mainen and Sejnowski, 1996). The passive electrical properties of the model were as follows: *R_m_* = 30,000 Ω cm^2^, *C_m_* = 0.75 μF cm^−2^, and *R_a_* = 150 Ω cm. The resulting somatic input resistance was 43 MΩ. ***B***, Basal dendrite region, which was sampled by moving a spine head and neck with passive properties sequentially between 117 different locations to test the role of the spine neck resistance *R*_neck_. ***C***, With the test spine at a single dendritic location, *R*_neck_ increases the spEPSP and values depend on the maximal synaptic conductance *G*_syn_ (right side color bar for families of curves). ***D***, At the same time, attenuation ratio of spine–somatic EPSP amplitude appears very linear and insensitive to *G*_syn_. ***E***, For each *R*_neck_ value, EPSPs were generated at all 117 basal dendritic locations, and the spine–soma attenuation ratio was plotted versus the distance of each model site (open circles) along with a quadratic fit (blue line) and superimposed with the experimental attenuation values (*N* = 19, closed circles with error bars). ***F***, For each experimental attenuation data point (distance, spine–soma attenuation), the model’s *R*_neck_ value that led to the quadratic fit (blue line) coinciding with that point was determined and plotted in a histogram (mean 179 ± 25 SE MΩ).

By adding a single spine to a biophysical neuronal dendrite model, we can study the effects of altering the spine neck resistance (Fig. 7). For spines with purely passive properties and an alpha-like synaptic conductance with a time course similar to our experimental data (τ_rise,_ τ_fall_ = 0.5, 4 ms), varying the neck resistance has the following effects. First, in the spine itself, EPSPs increase dramatically with increasing *R*_neck_ (Fig. 7*C*). The saturating nonlinearity comes from the reduction in driving force as spine signals become larger. Interestingly, as demonstrated in Figure 7*D*, the attenuation ratio between spine and soma is linearly related to *R*_neck_ even while spine and soma signals, individually, are in a nonlinear regime; furthermore, there is very little dependence of this attenuation on the size of the synaptic conductance, *G*_syn_.

Given the apparent power of attenuation ratios to predict *R*_neck_ and their insensitivity to *G*_syn_ (Fig. 7*D*), we used a biophysical model to estimate *R*_neck_ values that can reproduce our experimental attenuation data. Even though *G*_syn_ plays almost no role in EPSP attenuation, other factors, such as dendritic morphology, spine distance, etc, do play an important role along with *R*_neck_. For this reason, we used a previously published biophysical model and sampled the basal dendritic tree by moving the spine around to many different locations over all branches (*N* = 117 locations; Fig. 7*B*). At each location, we ran a series of *R*_neck_ values and found the *R*_neck_ value that fit each of our experimental attenuation ratio data points. For each *R*_neck_ test value, model attenuation ratio versus distance was fit well with an almost pure quadratic function (Fig. 7*E*). Notice that the model shows very little variability in attenuation especially for proximal distances, which supports our previous assertion that the large variability seen in the experimental data (clear ∼50 µm), must be due to a large variability in the *R*_neck_. This is also supported by the large variability we found in FRAP experiments (Fig. 6*E*). Fitting all experimental attenuation data points resulted in a distribution of estimated *R*_neck_ values (Fig. 7*F*) with mean 179 ± 25 SE MΩ (range, 23–420 MΩ, *N* =19).

We checked for sensitivity to several important model parameters. Increasing or decreasing sodium, potassium conductances by a factor of 2 led to small changes in mean predicted *R*_neck_ not >5%. Decreasing and increasing the passive membrane resistance by a factor of 2 changed predictions by −10.3 and 4.2%, respectively, with intracellular resistivity changing the result to a similar degree, but in the opposite direction as expected. Changing the rising phase of the synaptic conductance also changed the predicted *R*_neck_ values: factor of 2 changes led to modest changes of 10.8 or 21.0% depending on the direction.

Together, although results are relatively insensitive to several parameters, the model’s dendritic morphology, which can strongly alter the attenuation of potentials propagating to the soma (Rall and Rinzel, 1973; Carnevale and Johnston, 1982), remains a crucial assumption in our conversion of experimental attenuation ratios to predicted *R*_neck_ values. However, there is an excellent agreement between the model-dependent predicted values (mean 179, median 210 MΩ) and those obtained from an independent method using FRAP (Fig. 6; mean 204, median 161 MΩ; Table 1).

## Discussion

The electrical signal evoked in dendritic spines is arguably the most fundamental unit of information in the brain, but its amplitude and duration have been elusive (Yuste, 2013). In this study, we combined two-photon glutamate uncaging with two-photon voltage-sensitive dye recording to directly measure the characteristics of EPSPs in spines on basal dendrites of cortical L5 pyramidal neurons. We investigated their amplitude and duration in the spine, as well as their attenuation at the soma. To calibrate the voltage sensitivity individually in each spine, we measured the fluorescence change produced by a back-propagating action potential. These voltage-sensitive dye measurements of EPSPs were input to a biophysical model in order to estimate the spine neck resistance (*R*_neck_). Finally, FRAP measurements were used to produce an independent estimate of *R*_neck_. Both methods produced similar results with *R*_neck_ distributed at ∼180 MΩ.

Glutamate uncaging has been widely used to evoke single-spine EPSPs that resemble physiological synaptic activation (Matsuzaki et al., 2001). Such single events are usually <1 mV in amplitude at the soma for these cells (Williams and Stuart, 2002). Although glutamate uncaging is an artificial way to mimic synaptic glutamate release, it offers the best opportunity to study how electrical signaling from a single spine affects the electrical signaling at the soma. This is because electrical stimulation cannot be reliably targeted to activate only single spines. In our protocol, we tuned the position and intensity of the uncaging light (Fig. 1) to activate single spines with responses of <1 mV at the soma. The durations of these EPSPs in the spines had a mean of 11.7 ± 4.3 ms, significantly faster than somatic EPSPs. The kinetics of spEPSPs also contrast with glutamate uncaging-evoked spine Ca^2+^ signals that typically last ∼30 ms or longer (Yuste et al., 2000; Bloodgood and Sabatini, 2007).

We succeeded in measuring uncaging-evoked spEPSP amplitudes across spines of different sizes and morphologies. Although more data are needed to capture the full biological variability, these amplitudes ranged between 6.5 and 30.8 mV (Figs. 4, 5), with an average of 13.0 mV. The average amplitude ratio that we measured between the spine and the soma for these EPSPs is 25.3 ± 12.2. There was no correlation between this amplitude ratio and the distance between the spine and the soma (Fig. 5*D*), but a correlation could be expected if we had recorded from more spines at more distal locations. In addition to 20 spEPSP’s above the 2.5 × SD threshold that we used for reliable measurements, we also reported upper limits on an additional 28 spines where the S–N was too low to meet our threshold criterion, but for which we could measure somatic EPSPs and for which we could measure bAPs from the VSD fluorescence. This group had upper limits that fell within the range of 3.8–16.4 mV. Together these data make a good case for spEPSP amplitudes that are generally too low to evoke additional voltage dependent channel activity. In models, amplitude ratios across basal branches were well fit with an almost pure quadratic function with constant offset, which explains why we saw little correlation at proximal locations in experiments. Interestingly, variability calculated across branches in the model (Fig. 7*E*, blue circles) was very low for a fixed value of *R*_neck_. This suggests the high variability observed experimentally was likely due to the resistance of the spine neck, which is highly variable from spine to spine, as seen in FRAP experiments (Fig. 6*E*).

The amplitude of the uncaging-evoked spEPSP following stimulation of a single spine has been previously estimated from calcium measurements (Bloodgood et al., 2009; Harnett et al., 2012) or inferred from VSD measurements following synaptic stimulation of multiple synapses (Palmer and Stuart, 2009). We believe that VSD measurements confined to single spines, as shown in Figure 4, are arguably (Yuste, 2013) the most direct measurements of the amplitude and kinetics of these most fundamental neurophysiological signals. Our results partially support previous findings based on calcium imaging measurements, at least qualitatively: EPSPs in spines can be sufficiently large (up to 31 mV here) to activate local voltage-dependent conductances in spines, and any depolarization-dependent activation depends on the resistance of the spine neck. However, such large spEPSPs were the exceptions in our data, with only 2 of 48 measurements showing spEPSPs >22 mV (Fig. 4*B*). Thus our results differ quantitatively from the much larger spine EPSP amplitudes and neck resistances estimated from the calcium measurements (Bloodgood et al., 2009; Harnett et al., 2012). However, those results were performed on pyramidal neurons in hippocampal slices, as opposed to our studies on basal dendrites of L5 cortical neurons. Our results are closer to those of Palmer and Stuart (2009) and the recent single photon VSD measurements by Popovic et al. (2015) who studied spines from somatosensory cortex.

The results presented here depend on the calibration of voltage-sensitive dye measurements of spines, which was done by converting somatic AP amplitudes to bAP amplitudes in spines using an average attenuation versus distance curve (Fig. 2). Because the attenuation is expected to be small for proximal spines, and the majority of recordings were from spines within 60 µm of the soma, this is not expected to play a large role. Assuming a steeper attenuation of bAPs as reported by others (Nevian et al., 2007) we recalibrated spEPSPs, which were always smaller due to the apparent increased sensitivity (30.2% per 100 mV on average instead of 22.9). spEPSP amplitudes were on average 8.8 mV rather than 13.0 mV, reducing mean attenuation ratio to 18.0 from 25.3.

Calibrating spine potentials according to bAPs relies on accurate somatic AP measurements, along with knowledge of the distance-dependent attenuation to determine the bAP amplitude in the dendrite at the location of the spine of interest. This then matches the amplitude in the spine because the spine neck does not inhibit bAP penetration into the spine, as has been experimentally verified (Popovic et al., 2014). Other calibration signals can be and have been used, such as hyperpolarizing voltage pulses (Palmer and Stuart, 2009). These can be allowed to reach steady-state making them an attractive option. However, these are much smaller signals and space-clamp limitations governed by morphology and passive membrane also lead to propagation uncertainty, and if ignored would lead to overestimated EPSP amplitudes. We looked at both bAPs and hyperpolarizing pulses (−25 mV) in spines along basal dendrites of three cells and did not see a difference in the distance-dependent signals (data not shown).

It is important to reiterate that our calibration uses a simple average picture of distance-dependent AP attenuation along basal dendrites. There could be significant dendrite-to-dendrite variability that could affect the actual attenuation. Another related property is the local dendritic input impedance has been shown to affect local synaptic inputs as part of a voltage-divider circuit with the spine neck resistance (Harnett et al., 2012). Local dendritic voltage measurements during synaptic inputs would be advantageous in overcoming effects of local variability. However, our current two-photon single-voxel recording technique is less sensitive to dendritic signals presumably because of increased background fluorescence from internal membranes. This weakness could potentially be overcome by adding recordings from neighboring spines to report the common dendrite potential.

Several recent studies employing a variety of approaches have estimated *R*_neck._ One using both FRAP and direct morphological analysis reported values at ∼56 MΩ (Tonnesen et al., 2014). Another, using primarily calcium imaging, reported ∼500 MΩ (Harnett et al., 2012). Both of these examined spines from area CA1 of the hippocampus. A recent wide-field one-photon study of spEPSPs from cortical neurons (Popovic et al. 2015), compared uncaging voltage transients in spines and adjacent dendrites, detecting very little difference; the authors therefore concluded that *R*_neck_ is negligible_._ With the aid of the biophysical model, we used the experimentally determined ratio of spEPSP–soEPSP to estimate *R*_neck_ values for individual spines (Fig. 7*F*). The values are distributed around a mean of 179 ± 25 SE. In a completely separate set of measurements, we used FRAP together with a novel image analysis approach to accurately determine the volume of the spine head, a critical parameter for accurate estimation of *R*_neck_; the distribution determined from FRAP experiments (mean 204 ± 21 SE MΩ; Fig. 6) was close to that determined by analysis of spEPSP–soEPSP. Thus, our *R*_neck_ values are internally consistent based on two very different experimental approaches. The value of ∼180 MΩ is in the middle of the broad range estimated from other measurements.

In our study of proximal basal dendrites of L5 cortical neurons, direct VSD measurements, a biophysical model and FRAP experiments all produce a self-consistent picture of the role of the spine neck resistance in compartmentalizing electrical signals following glutamtergic activation of individul spines. The results indicate that the resistance of the spine neck is an important determinant of the attenuation of the EPSP between the spine and the soma. We expect that these basic results would hold if studied using natural axonal transmitter release rather than exogenous transmitter application using uncaging. However, detailed kinetics may change and may also depend on the presynaptic origin of the input. These are ideal applications of fast VSDs and continued work will be needed to elucidate the biophysical basis for the size and shape of the spEPSP along the dendritic tree of individual neurons in different brain regions.

## Synthesis

The decision was a result of the Reviewing Editor Michael Hausser and the peer reviewers coming together and discussing their recommendations until a consensus was reached. A fact-based synthesis statement explaining their decision and outlining what is needed to prepare a revision is listed below. The following reviewers agreed to reveal their identity: Valentin Naegerl, Greg Stuart

The study deals with a fundamentally important issue: the amplitude and time course of the EPSPs in the spine head, which is crucial for understanding how spines compartmentalise electrical signals. Because spine voltages cannot be measured directly by electrophysiology, the authors developed a new technique based on the use of voltage-sensitive dyes (VSD) to record voltage deflections inside spine heads induced by 2P glutamate uncaging. In parallel, they recorded somatic voltages with a patch-clamp electrode and performed mathematical modeling to estimate spine neck resistances (Rneck), which they compared with estimates based on FRAP measurements. They illustrate the method using experiments on L5 cortical neurons in acute brain slices at room temperature.

The main finding in the paper is that EPSP-like events in spines are quite variable, and range from around 5 to 20 mV in amplitude (average 9 mV). Modeling the data gives spine neck resistances from less than 50 MOhms to greater than 300 MOhms, with an average of around 150 MOhms. Importantly, this estimate is consistent with dye diffusion FRAP experiments in spines from the same cells.

The reviewers and editors agreed that the approach developed by the authors is innovative and powerful. The measurements improve on what has been published previously in terms of sensitivity (10 times less trial averaging than in Palmer and Stuart 2009) and directness (optical recording of voltage signals rather than Ca2+ imaging, as used by Bloodgood et al 2009 and Harnett et al 2012). What makes their approach particularly notable is the potentially high accuracy and validity of the measurements compared with previous studies. However, the referees raised several concerns that will need to be addressed:

MAJOR COMMENTS:

1. The exactitude of the values of the spine voltages stands and falls based on the quality of the calibration measurements. Using back-propagating APs to calibrate the optical measurements may not be sufficiently precise, considering that APs are highly dynamic (a couple of ms) and large signals (10x larger than the signals to be calibrated). The data cloud in Fig. 2C suggests a linear relationship between the VSD and calibration signals (which makes sense), but which appears to have a large negative offset (which doesn't make any sense at all). Because it is crucial to get the numbers right, it is important that the calibration procedure is beyond reproach. A better way may be to use hyperpolarizing voltage steps in the soma for "backward calibration" under steady-state conditions. Alternatively, they could also try to measure the somatic voltage optically, which would allow for a very accurate and direct "forward calibration". This may very well be feasible with multi trial and voxel averaging.

2. The estimate of Rneck based on VSD and electrophysiological measurements should depend strongly on the local dendritic input resistance, which they do not know, but which probably varies a lot between different dendrites and along a given dendrite. With their optical approach they should be able to measure the voltage in the dendrite right underneath the spine, which would give a direct read-out of the voltage attenuation by the spine neck and the neck resistance. Again, multi trial and voxel averaging should make this possible.

3. The study relies on 2P uncaging, which can produce voltage signals in the soma, which resemble EPSPs in amplitude and time course. However, the conductance change associated with synaptic release is likely to be very different (maybe 10 times faster in activation and deactivation?). Ideally one could record real EPSPs with their technique, using a minimal stimulation paradigm. The authors should mention this possibility and discuss this issue in the revised manuscript.

4. No attempt has been made to correlate the functional data sets (VSD and FRAP) with spine morphology. Fig. 6E is hinting at this, but this could be done more rigorously. Also, with their system, it should be possible to correlate the VSD with FRAP measurements in the same spine.

5. The Methods indicate that fluorescent voltage signals were filtered at 350 Hz and then passed through a 2.5 ms boxcar moving average. One might expect this filtering will impact on the amplitude of EPSP-like responses in spines. The magnitude of this effect could be tested using data obtained from simulations (assuming the simulations are realistic - see my next point). The Methods also indicate that a "0.5 ms uncaging artifact was removed from the optical recordings" (line 84). Again, the impact of this on the estimated amplitude of responses in spines could be tested using data from simulations.

6. The simulations use a synaptic conductance with an exponential rise time of 2 ms (line 189). Is this a typo? If not, this is very slow and unrealistic. More common would be 10-times faster (e.g. 0.2 ms time constant). It is also unclear whether spines not explicitly modeled were accounted for. As spines make up approximately half the surface area of dendrites with spines, their impact on passive membrane properties needs to be taken into account as this will have a significant impact on EPSP attenuation and therefore estimates of spine neck resistance based on the spine / soma attenuation ratio. To correct for this the membrane resistance is typically halved and capacitance doubled in dendritic segments with spines. Was this done?

MINOR COMMENTS:

1. The word "small" should be removed from the title. Estimation of whether something is small or big depends on what you comparing it to. In this case, relative to EPSPs at the soma, if anything, EPSP-like depolarizations in spines are big, not small (on average almost 20 times bigger, as indicated in the manuscript). The significance statement should similarly changed.

2. Given the variability it is be important to include the range of spine EPSP-like amplitudes and estimated spine neck resistances in the Abstract.

3. When averages are indicated in the text please indicate the number of observations.

4. In figures 1 and 3, please align (vertically) the electrical (somatic) and optical (spine) signals during AP and EPSPs separately. At the moment electrical (panel B) and optical signals (panel C) are aligned together, making comparison of electrical and optical signals difficult.

5. In the Discussion (line 52-55) it is suggested that larger estimates of EPSP amplitudes in spines by Bloodgood et al. (2009) and Harnett et al. (2012) can be explained by recruitment of voltage-dependent channels. We feel this is unlikely - while the signals recorded in these experiments relied on calcium influx through voltage-activated channels neither paper suggests that these channels amplify voltage responses.

6. It is not correct that the study by Palmer &amp; Stuart (2009) is "from the same brain region" (line 58). The experiments by Palmer &amp; Stuart (2009) are from somatosensory cortex.

7. The Methods indicate that slices were from the "frontal lobes" (line 35). Please be more specific as to the actual brain region.

8. Were the uncaging trials that did not lead to detectable transients included or rejected from the final average? What was this fraction?

9. The onset of synaptic voltage responses (real EPSPs) may be faster than during uncaging events due to better assess of synaptically released glutamate to postsynaptic receptors, leading to faster voltage changes in spines with higher amplitude. This possibility should be mentioned and discussed in the manuscript.

10. Lines 15, 17 and 18 in Materials and Methods: do they mean "emission channel"?

11. Line 140 in Results: FRAP
